# Canagliflozin attenuates isoprenaline-induced cardiac oxidative stress by stimulating multiple antioxidant and anti-inflammatory signaling pathways

**DOI:** 10.1038/s41598-020-71449-1

**Published:** 2020-09-02

**Authors:** Raquibul Hasan, Shoumen Lasker, Ahasanul Hasan, Farzana Zerin, Mushfera Zamila, Faizul Islam Chowdhury, Shariful Islam Nayan, Md. Mizanur Rahman, Ferdous Khan, Nusrat Subhan, Md. Ashraful Alam

**Affiliations:** 1 Department of Pharmaceutical Sciences, College of Pharmacy, Mercer University, 3001 Mercer University Drive, Atlanta, GA 30341 USA; 2 Department of Pharmaceutical Sciences, North South University, Bashundhara, Dhaka 1229 Bangladesh

**Keywords:** Biochemistry, Molecular biology

## Abstract

The antidiabetic drug canagliflozin is reported to possess several cardioprotective effects. However, no studies have investigated protective effects of canagliflozin in isoprenaline (ISO)-induced cardiac oxidative damage—a model mimicking sympathetic nervous system (SNS) overstimulation-evoked cardiac injuries in humans. Therefore, we investigated protective effects of canagliflozin in ISO-induced cardiac oxidative stress, and their underlying molecular mechanisms in Long-Evans rat heart and in HL-1 cardiomyocyte cell line. Our data showed that ISO administration inflicts pro-oxidative changes in heart by stimulating production of reactive oxygen species (ROS) and reactive nitrogen species (RNS). In contrast, canagliflozin treatment in ISO rats not only preserves endogenous antioxidants but also reduces cardiac oxidative stress markers, fibrosis and apoptosis. Our Western blotting and messenger RNA expression data demonstrated that canagliflozin augments antioxidant and anti-inflammatory signaling involving AMP-activated protein kinase (AMPK), Akt, endothelial nitric oxide synthase (eNOS), nuclear factor erythroid 2-related factor 2 (Nrf2) and heme oxygenase-1 (HO-1). In addition, canagliflozin treatment attenuates pro-oxidative, pro-inflammatory and pro-apoptotic signaling mediated by inducible nitric oxide synthase (iNOS), transforming growth factor beta (TGF-β), NADPH oxidase isoform 4 (Nox4), caspase-3 and Bax. Consistently, canagliflozin treatment improves heart function marker in ISO-treated rats. In summary, we demonstrated that canagliflozin produces cardioprotective actions by promoting multiple antioxidant and anti-inflammatory signaling.

## Introduction

Canagliflozin, a sodium-glucose cotransporter-2 (SGLT2) inhibitor, belongs to a new class of antidiabetic drugs prescribed for the management of type 2 diabetes mellitus (T2DM) ^1^. Accumulating evidence suggests that canagliflozin exhibits a range of cardiovascular effects that are independent of glucose lowering. According to recent preclinical and clinical data, canagliflozin treatment significantly reduced risk of cardiovascular death, myocardial infarction (MI), stroke and hospitalization due to heart failure in both diabetic and non-diabetic subjects ^2–5^. Proposed mechanisms for cardiovascular benefits of canagliflozin include improvement of cardiac metabolism and diastolic function, reduction of vascular stiffness, and an overall reduction of blood pressure ^5–9^. A growing body of evidence suggests that canagliflozin possess antioxidant and anti-inflammatory actions in various cell-based and animal models ^10–14^. Canagliflozin was shown to reduce vascular inflammation and atherosclerosis by suppressing vascular cell adhesion molecule-1 (VCAM-1) and monocyte chemotaxis protein-1 (MCP-1) expression ^15^. Consistent with its anti-inflammatory action, canagliflozin was demonstrated to reduce the production of inflammatory mediators such as interleukins and tumor necrosis factor-α in lipopolysaccharide-induced immune cells ^16^. Canagliflozin reduced cardiac nitro-oxidative stress, ameliorated cardiac ischemia–reperfusion injury (IRI), and improved heart function by stimulating antioxidant and anti-inflammatory signaling ^12,17^. Similarly, other members of SGLT2 inhibitors such as empagliflozin and dapagliflozin were also reported to improve cardiovascular function by reducing oxidative stress and inflammation ^10,14,18–21^.

Sympathetic nervous system (SNS) activity is essential for cardiovascular homeostasis, but SNS hyperactivity is associated with the development and progression of cardiovascular disorders including cardiac oxidative stress and inflammation, leading to cardiac remodeling, infarction and failure ^22–25^. SNS hyperactivity-associated β1 adrenergic receptor (β1-AR) overstimulation in heart promotes cardiac hypertrophy ^25,26^. On the other hand, persistent stimulation of β2-AR causes NADPH oxidase-induced ROS generation and inflammation leading to necrosis, fibrosis and MI ^22,24,27^. Importantly, there is a high prevalence of SNS hyperactivity in diabetic patients, which makes them more vulnerable to cardiovascular diseases. Unfortunately, options for treating SNS hyperactivity in diabetes are also limited as beta blockers, which are commonly used to treat SNS hyperactivity, are contraindicated in diabetes due to a range of life-threatening cardiovascular adverse effects ^28^.

**Figure 1:**
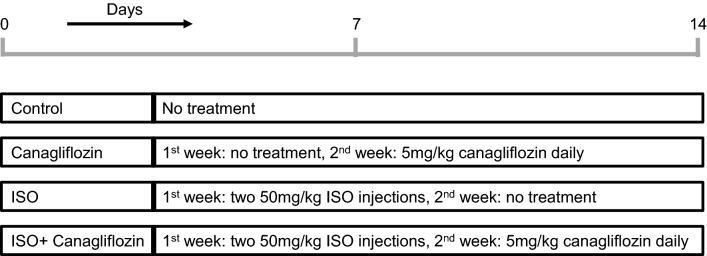
Schematic illustration of the experiment design. At the end of the 2-week experimental period, animals were euthanized, and tissues harvested for biochemical, histological and molecular analyses.

Since oxidative stress and inflammation are intricately associated with diabetes and other cardiovascular disorders, antidiabetic drugs with proven in-vivo antioxidative actions are expected to reduce the overall risk of cardiovascular events in diabetic patients, a viewpoint stated in a recent review article by Carbone and Dixon ^3^. Recent studies showing in-vivo antioxidant, anti-inflammatory and cardioprotective actions of canagliflozin raise the possibility that canagliflozin may be clinically useful in cardiac oxidative damage including that caused by SNS hyperactivity. Therefore, in the present study we first induced a model of chronic SNS overstimulation by administering the non-selective β-AR agonist isoprenaline (ISO) that produced profound cardiac injuries via ROS/RNS generation, inflammation and apoptosis ^22,24,25^, and then investigated protective effects of canagliflozin and their underlying molecular mechanisms. We found that canagliflozin augmented heart function marker in ISO-treated rats by stimulating multiple antioxidant and anti-inflammatory signaling pathways involving AMPK, Akt, eNOS, Nrf2 and HO-1 to counter ISO-induced oxidative damage caused by increased iNOS, TGF-β, Nox4, Bax and active caspase-3. Our study unveils a previously unrecognized role of canagliflozin in attenuating ISO-induced oxidative cardiac injuries and lends additional support to cardioprotective actions of canagliflozin reported previously. This study may open avenues for new pharmacotherapeutic indication of canagliflozin, particularly in patients with SNS hyperactivity (Fig. 1).

## Results

### Canagliflozin attenuates ISO-induced oxidative stress

**Figure 2:**
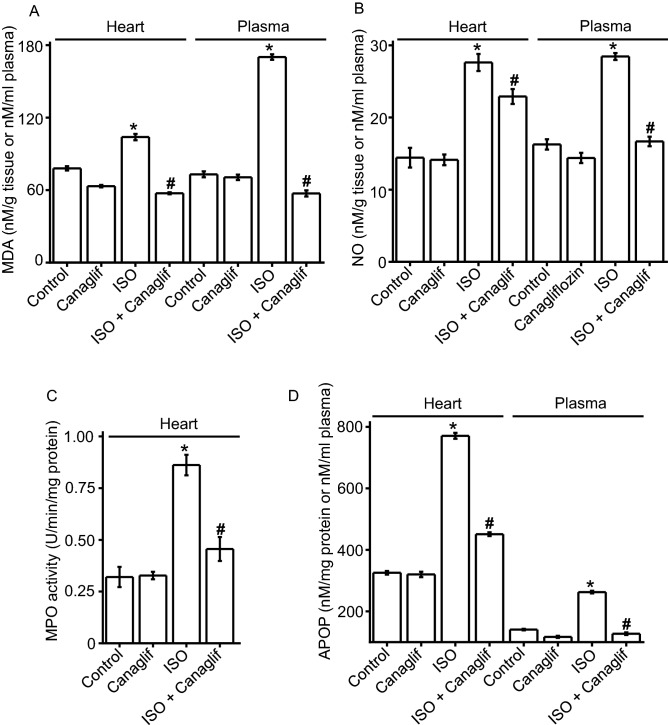
Canagliflozin treatment reduces ISO-induced elevation of oxidative stress markers in rat heart and plasma. One-way ANOVA with Newman–Keuls post hoc test was used for multiple group comparisons. Data are presented as mean ± SEM. n = 6 for each group. *Indicates p < 0.05 vs control, ^#^indicates p < 0.05 vs ISO.

Oxidative damage results from increased production of reactive oxygen species and/or decreased expression of endogenous antioxidant molecules ^29^. Since ISO has been reported to elevate reactive oxygen species (ROS), reactive nitrogen species (RNS) and deplete cellular antioxidants ^22,24,25^, we tested if canagliflozin can reverse such oxidative effects of ISO. Our data showed that ISO caused significant increases in the levels of oxidative and nitrative stress markers malondialdehyde (MDA), advanced protein oxidation product (APOP), myeloperoxidase (MPO) and excessive nitric oxide (NO) in heart tissue homogenates (Fig. 2A–D). Plasma levels of these markers also increased proportionately, reflecting tissue damage and leakage of the markers into the plasma (Fig. 2A,B,D). Note that due to the presence of several confounding factors such as hemolysis, heme-associated proteins and peroxidases that modulate MPO in the plasma, plasma MPO activity was not measured. Importantly, canagliflozin treatment significantly reduced ISO-associated rise of MDA, NO, MPO and APOP levels in heart (Fig. 2A–D) as well as in plasma (Fig. 2A,B,D), indicating that canagliflozin has in-vivo antioxidant action that can neutralize free radicals, ROS and RNS, and block their damaging effects on various cellular components.

### Canagliflozin preserves levels of endogenous antioxidants

**Figure 3:**
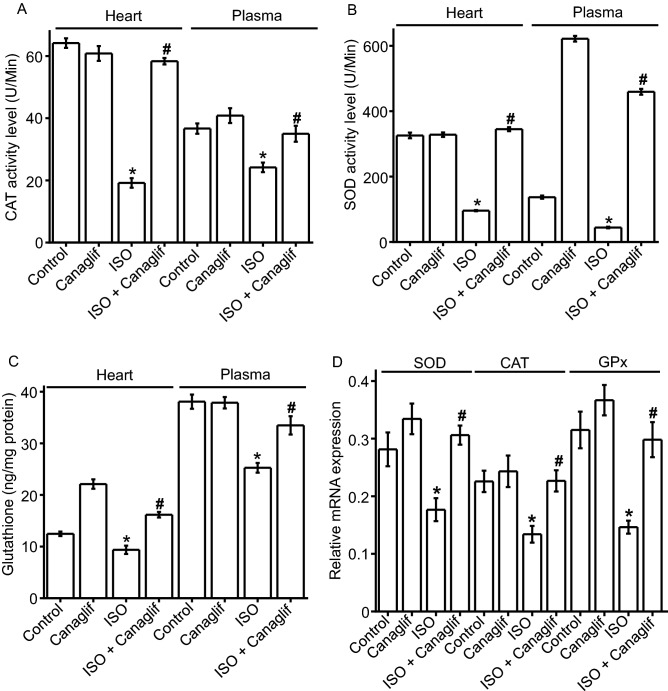
Canagliflozin prevents ISO-induced reduction of endogenous antioxidants in rat heart tissues and plasma. Statistical significance was measured using one-way ANOVA with Newman-Keuls post hoc test for multiple group comparisons Data are presented as mean ± SEM. n = 6 for each group. *Indicates p < 0.05 vs control, ^#^indicates p < 0.05 vs ISO.

Next, we examined effects of canagliflozin on the abundance and/or the activity of catalase (CAT), superoxide dismutase (SOD) and glutathione (GSH)—important endogenous antioxidant defenses against oxidative cellular injury. We found that in ISO-treated rats, CAT and SOD antioxidant enzyme activities in the plasma and heart tissue homogenate were significantly (p < 0.05) lower than those in corresponding controls (Fig. 3A,B). Similarly, concentrations of glutathione were significantly (p < 0.05) lower in ISO-treated rat plasma and heart tissue samples compared to controls (Fig. 3C). Consistent with previous data, canagliflozin treatment preserved CAT and SOD activities, and prevented glutathione reduction in plasma and heart tissue homogenate that was seen in ISO-treated rats (Fig. 3A–C). Using RT-PCR, we next measured mRNA levels for SOD, CAT and glutathione peroxidase (GPx) to determine if enzyme activity levels correlate with mRNA expression data for these antioxidant enzymes. As expected, ISO decreased and canagliflozin increased SOD, CAT and GPx mRNA expression (Fig. 3D), which is consistent with the enzyme activity data for SOD and CAT as shown in Fig. 3A,B. Taken together, these data suggest that canagliflozin boosts endogenous antioxidant defense by restoring cellular levels of the antioxidant enzymes both at mRNA and protein levels, which may contribute to the observed antioxidant action of canagliflozin in-vivo. Canagliflozin-mediated reduction of oxidative stress markers and maintenance of endogenous antioxidants in plasma are consistent with systemic antioxidant action of this drug beyond its local effects on cardiac tissues.

### Canagliflozin ameliorates ISO-induced pathological changes in heart

**Figure 4:**
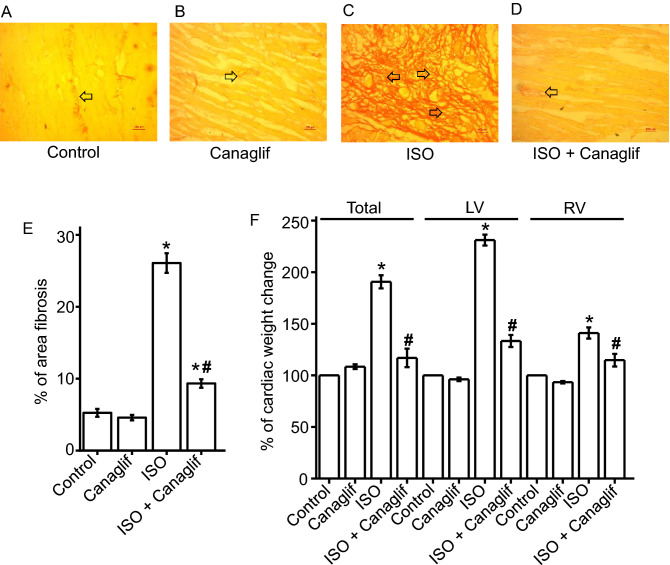
Canagliflozin (Canaglif) attenuates ISO-induced cardiac damage in rats. (**A**–**D**) Representative images of picrosirius red staining showing presence and extent of fibrosis in heart tissue sections under different conditions. (**E**) Mean data for % of area fibrosis, n = 12 for each group. (**F**) Mean data for cardiac hypertrophy showing % total heart, left ventricle (LV) and right ventricle (RV) weight changes compared to control. One-way ANOVA with Newman-Keuls post hoc test was used to identify significant differences. Data are presented as mean ± SEM. n = 6 for each group. *Indicates p < 0.05 vs control, ^#^indicates p < 0.05 vs ISO.

Since cardiac oxidative stress often leads to fibrosis, we tested the hypothesis that canagliflozin may protect cardiac tissue by reducing inflammation and fibrosis resulting from ISO-mediated ROS/RNS generation. Therefore, we evaluated effects of canagliflozin by picrosirius red staining on heart sections to visualize presence and extent of fibrosis. Our picrosirius red staining of Control and Canagliflozin rat heart tissue sections showed normal collagen distribution and alignments, indicating an absence of fibrosis (Fig. 4A,B,E), which was in contrast to those of ISO-treated rats, which exhibited excessive collagen deposition and fibrosis (Fig. 4C,E). Canagliflozin treatment significantly lowered collagen deposition in heart (Fig. 4D,I). Next, we examined the effect of canagliflozin treatment on ISO-induced cardiac hypertrophy and cardiac weight increases. As expected, ISO treatment produced a dramatic increase in total heart weight and left ventricular weight, indicating prominent left ventricular hypertrophy. Canagliflozin treatment reversed ISO-induced weight increase for total heart, left ventricle (LV) and right ventricle (RV) (Fig. 4F). This data suggests that canagliflozin is effective in reducing ISO-induced cardiac hypertrophy in rats.

### Effect of canagliflozin treatment on Nrf2 and TGF-β signaling

**Figure 5:**
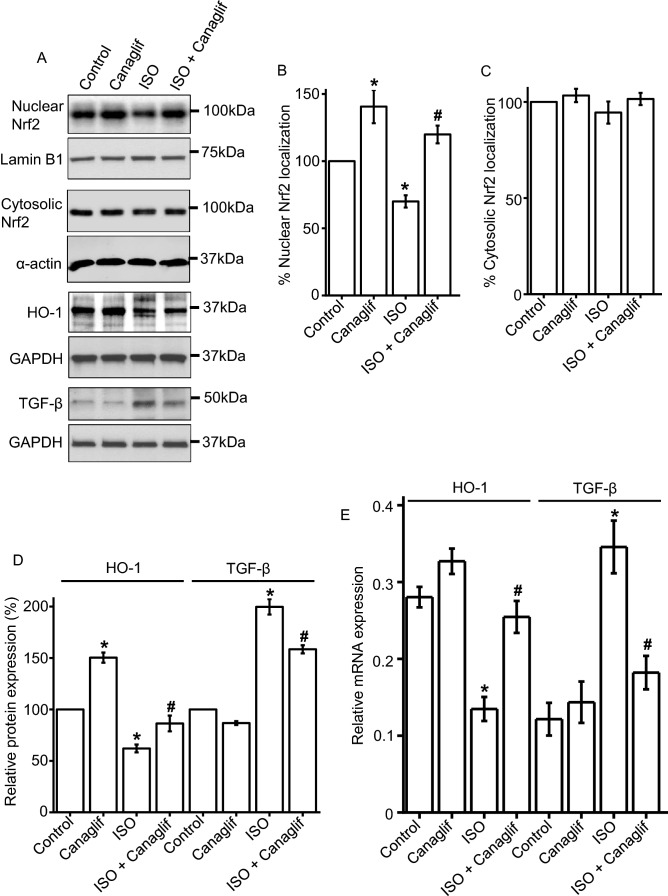
Canagliflozin stimulates Nrf2 nuclear translocation and HO-1 upregulation, and suppresses TGF-β in rat cardiac tissues. (**A**) Representative Western blot images of Nrf2 nuclear localization, HO-1 and TGF-β protein expression in rat heart tissues. (**B**) Mean data comparing relative changes of nuclear Nrf2 protein expression normalized to nuclear membrane marker Lamin B1. (**C**,**D**) Mean data comparing relative changes of HO-1 and TGF-β protein expression normalized to GAPDH or α-actin. n = 4 for each group. Blots were cropped to allow simultaneous probing of multiple proteins and full-length Western blot images are presented in Supplementary Fig. S1. (**E**) Mean quantitative real-time PCR data for mRNA transcripts of HO-1 and TGF-β normalized to β-actin mRNA from rat heart tissues. n = 4 for each group. One-way ANOVA with Newman-Keuls post hoc test was used for multiple group comparisons. Data are presented as mean ± SEM *indicates p < 0.05 vs control, ^#^indicates p < 0.05 vs ISO.

Nrf2 signaling protects against oxidative stress and inflammation, and heme oxygenase-1 (HO-1) is a Nrf2-regulated gene with prominent antioxidant, anti-inflammatory, anti-apoptotic, anti-proliferative and immunomodulatory effects ^30^. Therefore, we investigated the role of Nrf2 and HO-1 in ISO-induced oxidative stress. Our Western blotting data revealed that ISO treatment reduced nuclear translocation of Nrf2 and consequent HO-1 protein expression in rat cardiomyocytes, an effect that was reversed by canagliflozin treatment (Fig. 5A,B). Nrf2 protein levels in the cytosolic fractions were similar across different groups (Fig. 5A,C). This data suggests that canagliflozin treatment promotes nuclear translocation of Nrf2 and enhances the expression of HO-1 to elicit antioxidant and anti-inflammatory responses.

Since TGF-β is a potent cytokine involved in fibrosis as well as oxidative stress via stimulation of ROS production and suppression of antioxidant enzymes ^31^, we examined modulation of TGF-β expression by ISO alone and in combination with canagliflozin. We found that ISO caused ~ 2.15-fold increase in TGF-β protein expression, and such increase was significantly reduced by canagliflozin co-treatment (Fig. 5A,D). We also measured HO-1 and TGF-β mRNA expression to evaluate transcriptional modulation of HO-1 and TGF-β levels. Consistent with our protein expression analysis, RT-PCR data also showed that ISO treatment reduced HO-1 but enhanced TGF-β mRNA expression (Fig. 5E). As expected and consistent with its anti-inflammatory action, canagliflozin treatment rescued HO-1 mRNA expression, but suppressed TGF-β mRNA expression in ISO-treated rats (Fig. 5E). Altogether, these data indicate that canagliflozin treatment attenuates cardiac damage by stimulating Nrf2 and HO-1, and by inhibiting TGF-β.

### Canagliflozin stimulates phosphorylation of AMPK, Akt and eNOS to promote cardioprotective signaling

**Figure 6:**
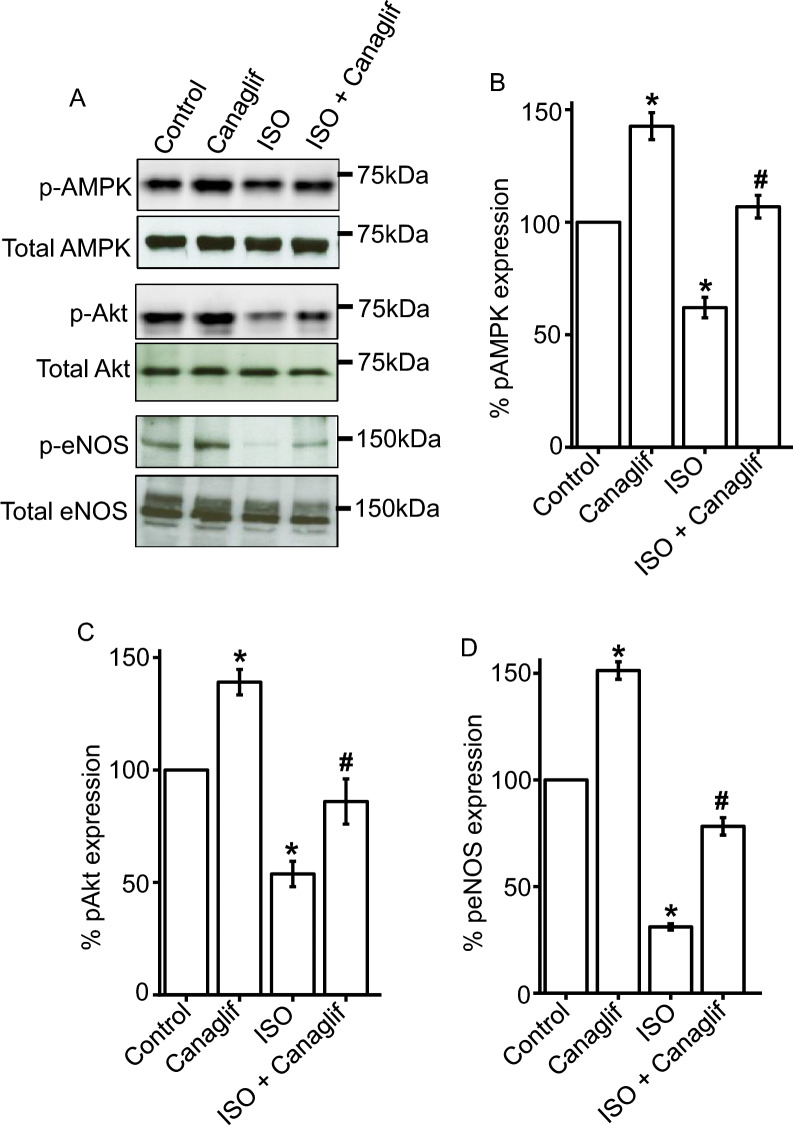
Canagliflozin stimulates AMPK, Akt and eNOS phosphorylation in rat cardiac tissues. (**A**) Representative Western blot images of phosphorylated (p) proteins normalized to their total protein expression for AMPK, Akt and eNOS. (**B**–**D**) Mean data comparing relative changes of AMPK, Akt and eNOS phosphorylation normalized to total protein expression. n = 4 for each group. Blots were cropped to allow simultaneous probing of multiple proteins and full-length Western blot images are presented in Supplementary Fig. S2. One-way ANOVA with Newman–Keuls post hoc test was used for multiple group comparisons. *Indicates p < 0.05 vs control, ^#^indicates p < 0.05 vs ISO.

Our data established protective roles of canagliflozin against ISO-induced oxidative stress in rat heart. Therefore, we tested if canagliflozin may stimulate AMPK, Akt and eNOS—critical signaling pathways that were reported to underlie protective effects in various cardiovascular diseases including myocardial IRI and infarction ^12^. We found that ISO reduced AMPK phosphorylation at the Thr172 activation site, which was significantly reversed by canagliflozin treatment (Fig. 6A,B). Canagliflozin treatment alone in Control rats also caused a significant increase in AMPK phosphorylation (Fig. 6A,B). This finding is consistent with previous reports showing that canagliflozin and empagliflozin stimulate phosphorylation of AMPK in IRI-induced myocardial ischemia model ^12^ and in Zucker diabetic fatty rats ^18^, respectively. In addition, ISO attenuated phosphorylation of Akt-Ser473 and eNOS-Ser1177 activation sites, both of which were rescued in heart tissues of canagliflozin-treated animals (Fig. 6A,C,D).

**Figure 7:**
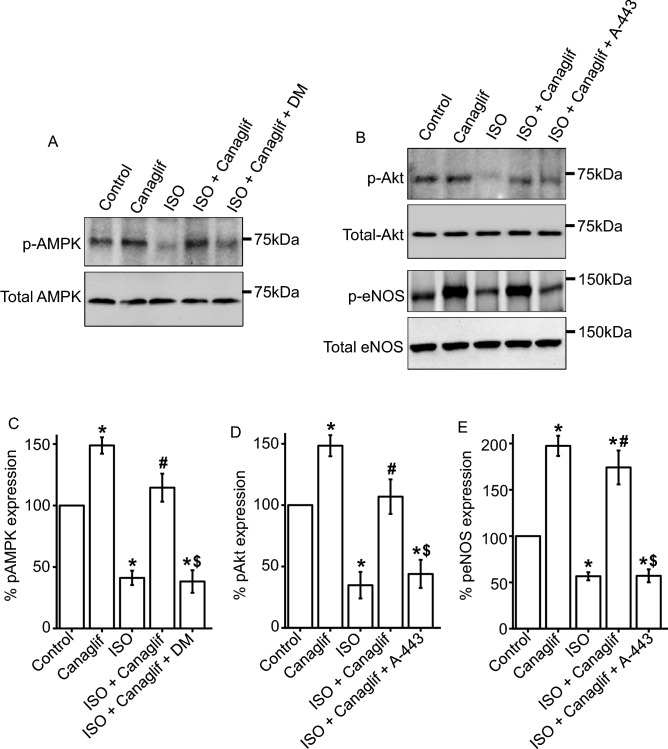
Canagliflozin stimulates AMPK, Akt and eNOS phosphorylation in HL-1 cardiomyocytes. (**A**,**B**) Representative Western blot images of phosphorylated (p) proteins normalized to their total protein expression for AMPK, Akt and eNOS. (**C**–**E**) Mean data comparing relative changes of AMPK, Akt and eNOS phosphorylation normalized to total protein expression. DM and A-443 indicate dorsomorphin and A-443654, respectively. Data are the mean of four individual experiments and expressed as mean ± SEM. Blots were cropped to allow concurrent probing of multiple proteins and full-length images are presented in Supplementary Fig. S3. One-way ANOVA with Newman-Keuls post hoc test was used for multiple group comparisons. *Indicates p < 0.05 vs control, ^#^indicates p < 0.05 vs ISO, $ indicates p < 0.05 vs ISO + Canaglif.

Using pharmacological inhibition, we further validated the involvement of AMPK, Akt, and eNOS signaling pathways in HL-1 cardiomyocytes. As expected and consistent with previous data, we found that ISO treatment reduced AMPK phosphorylation, which was reversed by canagliflozin treatment. However, application of dorsomorphin, an inhibitor of AMPK, completely abolished canagliflozin-mediated rescue of AMPK phosphorylation, further supporting the notion that canagliflozin indeed enhances AMPK phosphorylation (Fig. 7A,C). In addition, we found a similar modulation of Akt phosphorylation by ISO and canagliflozin, with ISO reducing and canagliflozin enhancing Akt phosphorylation, respectively. Importantly, Akt inhibitor A-443654 markedly attenuated canagliflozin-mediated enhancement of Akt phosphorylation (Fig. 7B,D). Since eNOS is a downstream target of Akt, we also examined the effect of Akt inhibition on eNOS phosphorylation. Our data showed that ISO reduced eNOS phosphorylation, and such reduction was reversed by canagliflozin treatment (Fig. 7B,E). However, application of A-443654 prevented canagliflozin-mediated reversal of eNOS phosphorylation (Fig. 7B,E). This finding indicates that activation of Akt is essential for downstream phosphorylation and activation of eNOS. These data in HL-1 cardiomyocytes lend additional support to our previous data showing the activation of AMPK, Akt, and eNOS signaling pathways in rat cardiac tissue following canagliflozin treatment. Altogether, our data demonstrated that canagliflozin enhances phosphorylation and activation of AMPK, Akt and eNOS, which may underlie cardioprotective actions of canagliflozin in ISO-induced oxidative stress (Fig. 11) ^12^.

### Canagliflozin suppresses ISO-induced Nox4 and iNOS upregulation

**Figure 8:**
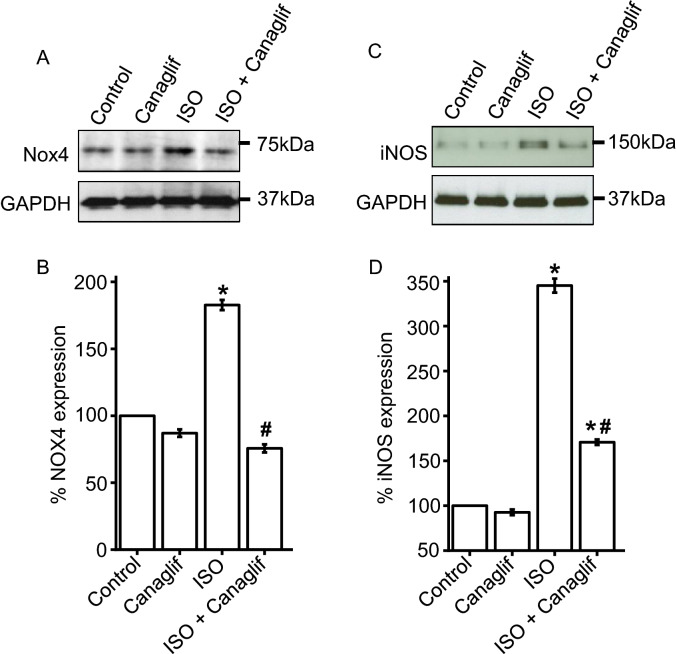
Canagliflozin reduces ISO-mediated upregulation of Nox4 and iNOS in rat heart. (**A**) Representative Western blot image illustrating a reduction of Nox4 protein expression by canagliflozin. (**B**) Mean data for changes of Nox4 expression normalized to GAPDH or α-actin. (**C**) Representative Western blot image showing a reduction of ISO-induced iNOS upregulation by canagliflozin treatment. (**D**) Mean data for changes of iNOS expression normalized to GAPDH or α-actin. n = 4 for each group. Data expressed as mean ± SEM. Blots were cropped to allow simultaneous probing of multiple proteins and full-length Western blot images are presented in Supplementary Fig. S4. One-way ANOVA with Newman-Keuls post hoc test was used for multiple group comparisons.*Indicates p < 0.05 vs control, ^#^indicates p < 0.05 vs ISO.

Previous studies showed that ISO-mediated β-AR overstimulation increases expression of Nox4 (NADPH oxidase isoform 4) at mRNA and protein levels ^22^. Our data also showed that ISO enhances expression of TGF-β, which is known to upregulate Nox4 ^31^. This prompted us to investigate potential involvement of Nox4 upregulation and oxidative stress in our model and evaluate the role of canagliflozin treatment in Nox4 upregulation. Our data showed that ISO-treatment resulted in ~ 1.8-fold increase in Nox4 protein expression (Fig. 8A,B), while canagliflozin treatment resulted in ~ 60% reduction of ISO-stimulated Nox4 overexpression in heart (Fig. 8A,B).

While eNOS-mediated production of NO is essential for cardiovascular function, excessive NO production by iNOS, which is often upregulated during inflammation, leads to nitrative stress and cellular injury. Again, since ISO treatment dramatically enhanced NO production (Fig. 2B), we investigated if iNOS was upregulated in ISO-treated rat tissues and can be modulated by canagliflozin treatment. Our Western blotting data show that iNOS expression is negligible in control rat heart tissues (Fig. 8C,D), but increased ~ 3.5-fold upon ISO treatment in heart tissue (Fig. 8C,D). Importantly, canagliflozin treatment led to ~ 50% reduction of iNOS protein levels in heart tissues of ISO-treated rats (Fig. 8C,D), suggesting a putative role of canagliflozin treatment in attenuating iNOS upregulation induced by ISO.

### Canagliflozin treatment attenuates ISO-induced cardiomyocyte apoptosis

**Figure 9:**
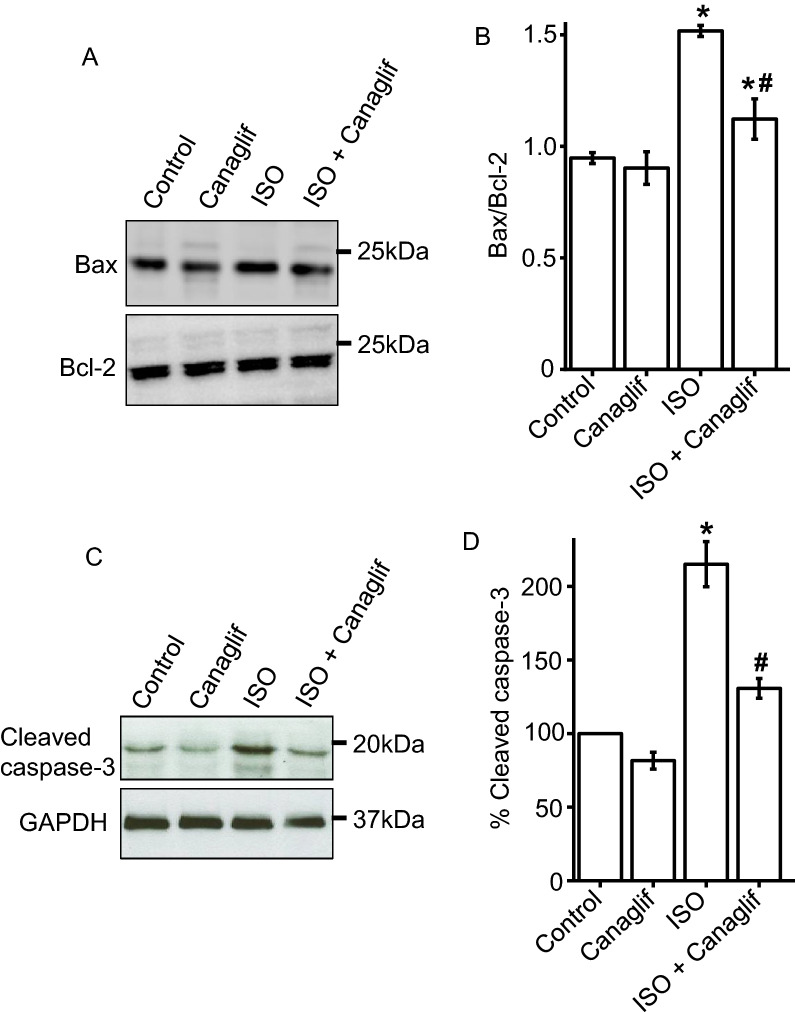
Canagliflozin reduces ISO-induced apoptosis of rat cardiomyocytes. (**A**) Representative Western blot image showing a reduction of Bax expression by canagliflozin. (**B**) Mean data for changes of Bax expression relative to anti-apoptotic protein Bcl-2 expression. n = 4 for each group. (**C**) Representative Western blot image showing a reduction of cleaved caspase-3 expression by canagliflozin. (**D**) Mean data comparing relative changes of cleaved caspase-3 expression. n = 4 for each group. Data presented as mean ± SEM. Blots were cropped for simultaneous probing of multiple proteins and full-length images are presented in Supplementary Fig. S5. One-way ANOVA with Newman–Keuls post hoc test was used for multiple group comparisons *indicates p < 0.05 vs control, _#_indicates p < 0.05 vs ISO.

Since AMPK and Akt activation are reported to reduce cell death associated with myocardial IRI ^12^, we asked if AMPK and Akt activation by canagliflozin might attenuate ISO-evoked oxidative damage and cell death in rat heart. Hence, we analyzed the ratio of pro-apoptotic Bax and anti-apoptotic Bcl-2 protein expression, and our data showed that the ratio of Bax and Bcl-2 protein expression in Control and Canagliflozin-treated animals were ~ 0.95 and ~ 0.90, respectively (Fig. 9A,B). Non-treated ISO rat heart had a Bax/Bcl-2 of ~ 1.51, representing ~ 60% increase of Bax/Bcl-2 ratio compared to controls. As expected, Canagliflozin + ISO treatment significantly reduced Bax/Bcl-2 ratio to 1.12 (Fig. 9A,B). To further validate the role of canagliflozin on cardiomyocyte viability under ISO-induced oxidative stress, we analyzed levels of another apoptotic marker, cleaved caspase-3. Cleaved caspase-3 is an active form of caspase-3 involved in apoptosis through its ability to degrade various cellular components, leading to DNA fragmentation, destruction of cytoskeletal proteins and cell death ^32^. Our data revealed that cleaved caspase-3 levels are low in tissues from Control and Canagliflozin groups (Fig. 9C,D), indicating low caspase-3 activity and apoptosis. In contrast, ISO treatment caused a dramatic increase in cleaved caspase-3 levels, indicating enhanced apoptosis of cardiomyocytes. Importantly, canagliflozin treatment suppressed ISO-induced elevation of cleaved caspase-3 expression (Fig. 9C,D), suggesting that canagliflozin is effective in reducing ISO-induced apoptosis of cardiomyocytes. In summary, these data led us to conclude that canagliflozin treatment attenuates ISO-mediated death of cardiomyocytes perhaps via modulation of AMPK and Akt signaling (Fig. 11).

### Canagliflozin reduces plasma levels of heart function marker in ISO-treated rats

**Figure 10:**
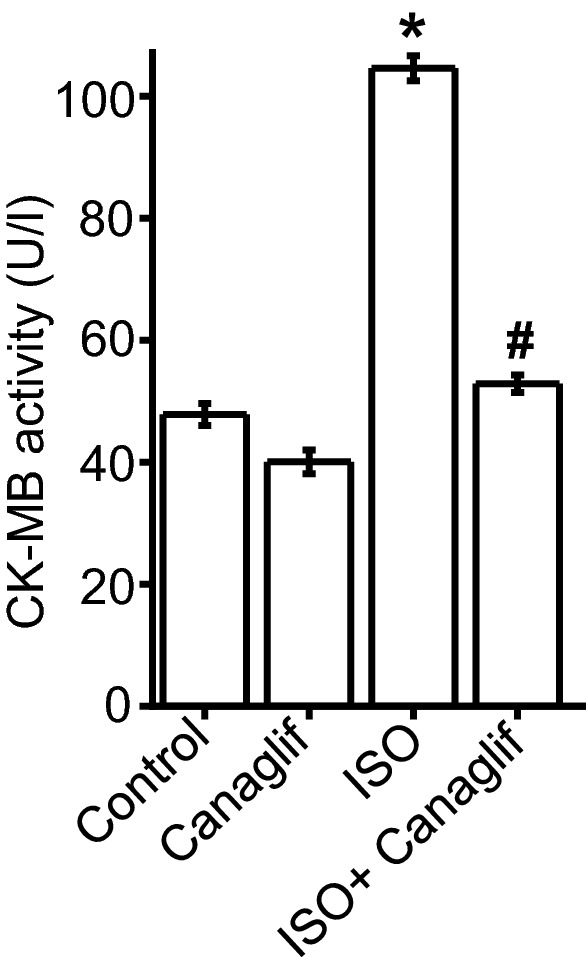
Canagliflozin treatment attenuates CK-MB activity in ISO-treated rats. Mean data showing that canagliflozin potently suppressed elevation of heart function marker CK-MB in the plasma. n = 6 for each group. Data presented as mean ± SEM. One-way ANOVA with Newman-Keuls post hoc test was used for multiple group comparisons *indicates p < 0.05 vs control, ^#^indicates p < 0.05 vs ISO.

**Figure 11:**
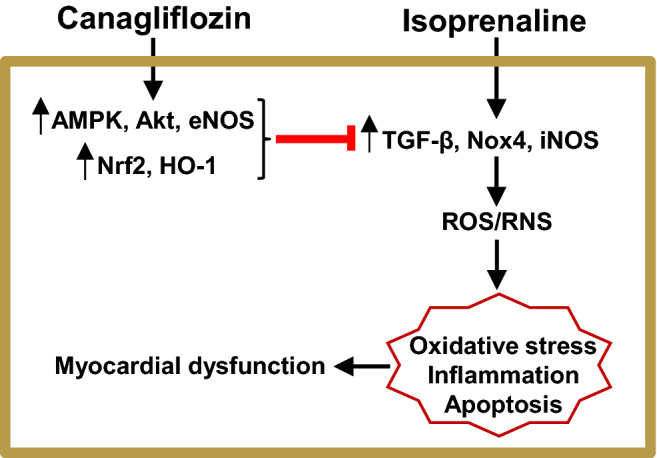
Schematic diagram illustrating proposed mechanisms of action of canagliflozin in reducing ISO-induced cardiac oxidative stress. Canagliflozin stimulates antioxidant, anti-inflammatory and anti-apoptotic signaling pathways involving AMPK, Akt, eNOS, Nrf2 and HO-1 and suppresses pro-oxidative, pro-inflammatory and pro-apoptotic signaling involving enhanced TGF-β, Nox4, iNOS.

Oxidative cardiac injury leads to increased plasma creatinine kinase muscle brain (CK-MB) activity. As canagliflozin exhibited potent antioxidant, anti-inflammatory and anti-apoptotic effects, we sought to determine the functional outcome of such actions on heart by analyzing CK-MB activity, an important marker for heart function. We found that ISO administration caused a ~ 2.6-fold increase in plasma CK-MB activity (Fig. 10), presumably due to oxidative damage to cardiomyocytes and subsequent leakage of CK-MB into the bloodstream. Canagliflozin treatment significantly suppressed plasma CK-MB rise by ISO (Fig. 10), indicating remarkable cardioprotective role of canagliflozin against oxidative injury. In summary, consistent with improved biochemical markers reflecting reduced oxidative stress and inflammation, canagliflozin treatment also improved heart function marker in ISO-treated rats by stimulating antioxidant, anti-inflammatory and anti-apoptotic signaling to counter deleterious effects of ROS/RNS produced by ISO (Fig. 11).

## Discussion

Our study for the first time shows that canagliflozin has cardioprotective actions in ISO-induced oxidative stress model which recapitulates pathological features of SNS overstimulation-mediated oxidative cardiac injury in humans ^22,23,33^. We show that in ISO-treated rats, canagliflozin significantly reduced cardiac oxidative stress markers, maintained endogenous antioxidants, reduced, fibrosis and apoptosis. We further show that canagliflozin augmented antioxidant/anti-inflammatory signaling that involves AMPK, Akt, eNOS, Nrf2 and HO-1, and attenuated pro-oxidative/pro-inflammatory and pro-apoptotic signaling involving iNOS, TGF-β, Nox4, Bax and active caspase-3 (Fig. 11). Consistent with a central protective action, canagliflozin treatment also improved heart function marker in ISO-treated rats.

It is well established that ISO stimulates β-AR, primarily β2-AR, to cause oxidative stress leading to inflammation and tissue damage ^22,27,34,35^. ISO-induced oxidative stress is characterized by elevation of various oxidative and nitrative stress markers including MDA, NO, MPO and APOP, with concurrent reduction of cellular reserves of naturally occurring antioxidants such as CAT, SOD and glutathione ^36,37^. Our data showed that canagliflozin potently reduced MDA, NO, MPO and APOP in heart tissue homogenate (Fig. 2). Canagliflozin also maintained levels of endogenous CAT, SOD and glutathione (Fig. 3A–C), which is consistent with our mRNA expression data for CAT, SOD and GPx (Fig. 3D). Our Western blotting data showing the ability of canagliflozin to strongly suppress ISO-mediated overexpression of Nox4, a key enzyme that produces superoxide radical leading to oxidative stress and inflammation, led us to propose that Nox4 reduction may partially account for antioxidant and anti-inflammatory actions of canagliflozin. ISO-treated rats further showed a dramatic upregulation of TGF-β (Fig. 5), which may induce oxidative stress by enhancing Nox4 expression and ROS production ^31^. Canagliflozin treatment suppressed TGF-β expression, leading us to propose that canagliflozin-mediated reduction of Nox4 expression may result from an inhibition of its upstream regulator TGF-β. However, whether canagliflozin acts directly on Nox4 or via TGF-β remains to be determined. Canagliflozin treatment also stimulated nuclear translocation of Nrf2 and upregulation of HO-1 protein (Fig. 5). Since HO-1 has strong antioxidant and anti-inflammatory effects ^30^, elevated HO-1 levels by canagliflozin treatment may reduce oxidative stress in ISO-treated rats. In general, our data appears to suggest multiple mechanisms, rather than a specific pathway, to underlie canagliflozin mediated antioxidant and anti-inflammatory actions (Fig. 11).

We also found that canagliflozin treatment significantly reduced expression of iNOS which, unlike eNOS, is exclusively upregulated during cardiac oxidative stress and inflammation ^38–40^. eNOS is known to protect against oxidative damages to the heart ^12,41^ through regulated NO production. In contrast, iNOS serves as a key mediator of nitro-oxidative stress by producing exorbitant amounts of NO that reacts with superoxide to form peroxinitrite ^38,42^. In our study, ISO-mediated cardiac oxidative stress in ISO-treated rats may arise from iNOS induction and excessive NO generation, as well as Nox4 overexpression and superoxide production. In addition, ISO-treated rat tissues had significantly reduced SOD and CAT, which are involved in the removal of superoxide produced by various pathways including Nox4. Collectively, our data suggest that canagliflozin promotes antioxidant and anti-inflammatory actions by regulating expression of these key proteins involved ^12,43^. This finding agrees with previous studies showing that a closely related SGLT2 inhibitor empagliflozin has antioxidant and anti-inflammatory effects in heart as well as in blood vessels ^18–21^. In another report, empagliflozin was shown to reduce cardiac oxidative stress and fibrosis in diabetic mice by stimulating Nrf2/HO-1 pathway and suppressing TGF-β ^44^.

In addition to its inhibitory action against pro-oxidative signaling, canagliflozin also stimulated a well-recognized protective signaling axis involving AMPK, Akt and eNOS in both rat heart tissues as well as in HL-1 cardiomyocytes. We found that canagliflozin treatment markedly enhanced phosphorylation and activation of AMPK (Figs. 6, 7), a master regulator of energy homeostasis ^45^. Several recent studies also demonstrated that canagliflozin activates AMPK in cardiomyocytes ^12^, hepatocytes ^46^ and endothelial cells ^34^ to produce a range of beneficial effects in the cardiovascular system and beyond ^47^, including anti-inflammatory actions and protection against myocardial ischemia ^12^. Canagliflozin was reported to enhance phosphorylation of Akt, which was shown to provide cardioprotection during myocardial IRI ^12^. Activated AMPK and Akt phosphorylate eNOS-Ser1177 activation site, which in turn stimulates NO production. Previous studies demonstrated that preserved eNOS-mediated NO signaling reduces oxidative stress, apoptosis, and platelet aggregation in ischemic heart following canagliflozin administration ^12,40^. AMPK-Akt activation was also implicated in apoptosis inhibition following myocardial IRI ^12,48,49^. In a recent study, Aragon-Herrera et al. showed that empagliflozin stimulates cardiomyocyte AMPK to induce autophagy, which was shown to improve heart function by enhancing cardiac energy metabolism in Zucker diabetic fatty (ZDF) rats ^18^. However, it is beyond the scope of our study to determine if canagliflozin would have similar actions in our model of ISO-induced oxidative stress. Our data showed that canagliflozin suppressed pro-apoptotic protein Bax such that the ratio of Bax and Bcl-2 protein expression ≤ 1.0 to favor cardiomyocyte viability. Canagliflozin treatment also strongly suppressed caspase-3 activation as evidenced by reduction of cleaved caspase-3 levels (Fig. 9C,D). Therefore, the overall reduction of ISO-mediated oxidative stress, fibrosis and apoptosis of cardiomyocytes by canagliflozin is likely due to combined effects of preserved NO signaling, AMPK-Akt activation as well as reduction of Bax, Nox4, iNOS, and caspase-3 activation. Our data also demonstrated that canagliflozin stimulated nuclear translocation of the transcription factor Nrf2 to upregulate its downstream target HO-1 that has anti-apoptotic action, in addition to its antioxidant, anti-inflammatory effects ^30^. Behnammanesh et al. demonstrated that in rat and human aortic smooth muscle cells, canagliflozin stimulates Nrf2 activation and downstream HO-1 induction in a ROS-dependent manner ^11^. Based on this study, it is possible that canagliflozin initially stimulates ROS production in rat cardiomyocytes, causing Nrf2 activation and HO-1 overexpression. However, following HO-1 induction, the antioxidant actions of HO-1 may neutralize the formed ROS and shift the balance of oxidative stress towards normal via a negative feedback mechanism. Our data showed that, apart from HO-1-mediated antioxidant action, canagliflozin also stimulates several other antioxidant defenses via the activation of AMPK-Akt-eNOS axis and inhibition of iNOS and NOX4. This may explain the enhanced nuclear translocation of Nrf2 and HO-1 induction in canagliflozin-treated rat heart tissues, which contributes to the overall antioxidant action of this drug. Empagliflozin has also been reported to enhance Nrf2 activation and nuclear translocation in diabetic rat cardiomyocytes ^44^. Although the underlying mechanism(s) remains unclear, our data is in agreement with the above studies showing Nrf2 activation and HO-1 induction following canagliflozin treatment ^11,44^. These observations further reinforce the concept that multiple mechanisms and potentially additional mechanisms may be involved. Previous studies reported antioxidant and anti-inflammatory actions of canagliflozin and other SGLT2 inhibitors in IRI and chemically induced cardiac and renal oxidative stress ^12,50,51^. Unlike previous studies, our study for the first time demonstrated that canagliflozin exhibits antioxidant and anti-inflammatory actions in ISO-induced cardiac oxidative stress, an animal model that mirrors many pathological and morphological changes associated with SNS overstimulation-induced cardiac damage in humans ^22,23,33^. Given the prevalence of SNS overstimulation in diabetic patients and limited treatment options ^28^, canagliflozin monotherapy may have potential for the treatment of SNS hyperactivity in diabetic patients.

Our study showed that canagliflozin is effective in reducing plasma oxidative stress markers and preserving plasma levels of naturally occurring antioxidants such as CAT, SOD and glutathione. These findings support the notion that SGLT2 inhibitors may have systemic antioxidant/anti-inflammatory actions beyond its localized effects on cardiac tissues. Consistent with improved biochemical markers, canagliflozin treatment of ISO rats provided cardioprotection and prevented leakage of heart function marker CK-MB into the blood, presumably by stimulating antioxidant, anti-inflammatory and anti-apoptotic signaling. The primary antidiabetic action of canagliflozin is due to a reduction of glucose reabsorption via blockade of SGLT2, a glucose transporter having high abundance in kidneys and intestines but not in other tissues such as heart ^12,52^. Our data showed that canagliflozin reduced cardiac oxidative stress and improved cardiac function, suggesting that such effects are independent of organ specific SGLT2 expression, and likely to be pleiotropic ^12,52^.

Our data appears to suggest involvement of multiple pathways for antioxidant/anti-inflammatory actions of canagliflozin (Fig. 11). So, we cannot rule out the possibility that other molecular mechanisms, in addition to those described here, may also be responsible for the cardioprotective effects of canagliflozin. As diabetes is invariably associated with some degree of oxidative stress and inflammation of the cardiovascular system, we used an induced model of cardiac oxidative stress to investigate antioxidant potential of canagliflozin to avoid interference from other mechanisms specific for diabetes. Therefore, future studies in diabetic animal models will be required to understand full therapeutic potential of this drug in SNS overstimulation-induced oxidative stress and organ injuries.

In conclusion, our data demonstrated that canagliflozin has strong in-vivo antioxidant actions that prevented SNS hyperactivity-induced cardiac damage, and improved cardiac function marker. Such actions may involve multiple mechanisms (Fig. 11), extend beyond localized actions in heart to broad systemic effects, and likely involve other organs affected by SNS hyperactivity such as kidney, vasculatures and liver. Considering the prevalence of SNS hyperactivity in diabetic patients and complications associated with conventional beta blocker treatment, canagliflozin monotherapy may offer an attractive alternative in this scenario. As our study was conducted in non-diabetic animals, future studies on ISO-induced SNS hyperactivity in diabetic animals would be interesting to investigate.

## Materials and methods

### Chemicals and antibodies

Dimethyl sulfoxide (DMSO), ethanol, xylene, reduced glutathione, thiobarbituric acid, 10% neutral buffered formalin (NBF) solution, all primers, protease and phosphatase inhibitor cocktails, HL-1 cardiomyocyte cell line and all culture media components were purchased from Sigma Aldrich (St. Louis, MO, USA). Canagliflozin was purchased from both Sigma Aldrich (St. Louis, MO, USA) and Square Pharmaceutical Ltd. (Dhaka, Bangladesh), and isoprenaline (ISO) from Samarth Life Sciences Pvt. Ltd. (Mumbai, India). Standards and all other components for MDA, NO, APOP assays, and picrosirius red staining components were from Merck (Darmstadt, Germany). SOD standard and other assay components were purchased from SR Group (Delhi, India). Assay kits for CK-MB was purchased from DCI diagnostics (Budapest, Hungary). Cell fractionation kit was purchased from Cell Signaling Technology (Danvers, MA, USA). Dorsomorphin was purchased from Santa Cruz Biotechnology (Santa Cruz, CA, USA). Phosphate-buffered saline (PBS, pH 7.4), radioimmunoprecipitation (RIPA) buffer, blot stripping buffer (Restore™), 2X SYBR Green qPCR Master Mix, kits for RNA purification and cDNA synthesis were bought from Thermo Fisher Scientific (Waltham, MA, USA), and A-443654 from MedChemExpress (Monmouth Junction, NJ, USA). DC Protein Assay Kit, tween 20, SDS-PAGE gel components, protein molecular weight marker, 10X tris-buffered saline (TBS) and PVDF (polyvinylidene fluoride) membrane were bought from Bio-Rad (Hercules, CA, USA).

### Animals and experimental design

All animal protocols were approved by the Ethics Committee of North South University (AEC 005-2018). Experiments were conducted following the guidelines set by the United States National Institutes of Health Guide for the Care and Use of Laboratory Animals 24 male Long Evans rats, 10–12 weeks of age, obtained from the Reproduction unit of the Animal House at North South University, Dhaka, were used for this study. Animals were individually caged in a temperature- regulated room (temperature 22 ± 2 °C; 55% humidity; 12-h light/dark cycles) and all animals had access to standard chow diet and drinking water ad libitum. Animals were randomized into four groups of six rats in each and treated as follows:

Group I: Control—received standard chow diet for 2 weeks.

Group II: Canagliflozin—received standard chow diet for the first week and then received canagliflozin at 5 mg/kg daily with chow diet for the second week ^53^.

Group III: ISO—received subcutaneous injection of isoprenaline at 50 mg/kg twice a week for the first week. Animals in this group were placed on standard chow diet during the 2 weeks of experimental period.

Group IV: ISO + Canagliflozin—received subcutaneous injection of isoprenaline at 50 mg/kg twice a week for the first week and treated with canagliflozin at 5 mg/kg daily for the second week.

Body weight, food consumption and water consumption were recorded daily for 2 weeks. A schematic illustration of the experimental design and treatment protocol is shown in Fig. 1.

### Euthanasia and tissue harvesting

At the end of the 2-week experimental period, animals were euthanized with intraperitoneal injection of ketamine/xylazine (500/50 mg/kg) followed by decapitation. Blood was collected from the hepatic portal vein and plasma separated by spinning samples at 8,000 rpm for 15 min at 4 °C. Separated plasma was used either immediately for biochemical analysis or stored at − 80 °C for future experiments. Whole heart was collected, weighed and stored in neutral buffered formalin (pH 7.4) for histological analysis. Heart tissues were processed for Western blotting as well as for microscopic examination and the remaining tissue samples stored at − 80 °C for future analysis.

### Plasma biochemistry

Plasma creatinine kinase muscle brain (CK-MB) was determined using kits following manufacturer’s instructions (DCI Diagnostics, Budapest, Hungary).

### Determination of oxidative and nitrative stress markers: malondialdehyde (MDA), nitric oxide (NO) and advanced protein oxidation products (APOP)

Approximately 0.1 g of heart tissue was homogenized in 1 mL Phosphate buffer (pH 7.4) and centrifuged at 10,000 rpm for 15 min at 4 °C. The supernatant was collected and used for the determination of MDA, NO and APOP. Lipid peroxidation was quantified by estimating MDA levels in heart tissue homogenates by colorimetric assay ^54^. Determination of NO was performed by the method of Tracey et al. ^55^. NO level was calculated by using a standard curve and expressed as nmol/g of tissue. APOP levels were determined according to the modified method of Witko-Sarsat et al. ^56^ and Tiwari et al. ^57^. The chloramines-T absorbance at 340 nm is linear within the range of 0 to 100 mmol/L. APOP levels were expressed as nmol/mL chloramine-T equivalents.

### Estimation of myeloperoxidase (MPO) activity

MPO activity was determined by an *o*-dianisidine-H_2_O_2_ method modified for 96-well plates as described previously by Rahman et al. ^58^.

### Determination of endogenous antioxidant levels: estimation of catalase (CAT) and super oxide dismutase (SOD) activity and glutathione (GSH) levels

CAT activities in plasma and heart tissue homogenate were determined following protocols previously described ^47,59^. Absorbance changes were read at 240 nm. An absorbance change of 0.01 units/minute was interpreted as one unit of CAT activity. SOD activity was determined according to methods described previously ^47,59^. Reaction mixtures containing enzymes were prepared and absorbance read at 480 nm for 1 min at 15 s intervals. A blank without tissue homogenates was run in parallel. Auto-oxidation of epinephrine present in the assay system was calculated and 50% inhibition is defined as the one unit of SOD enzyme activity. Reduced glutathione level was estimated following protocols as described by Jollow et al. ^60^. With the development of yellow chromophore the mixture was read immediately at 405 nm in a UV–Vis spectrophotometer and glutahione level expressed as ng/mg protein.

### Histopathological examination

For the histopathological evaluation, isolated heart tissue sections of the experimental rats were initially fixed in 10% Neutral Buffered Formalin (NBF) followed by their treatment with graded ethanol and xylene. Tissue sections were subsequently embeded into paraffin blocks which were cut with a rotary microtome into 5 µm thin slices that were collected on fresh slides and stained with picrosirius red to analyze the presence and extent of fibrosis. After completing the staining procedure, all tissue section slides were photographed and analyzed under a light microscope at 40X magnification (Zeiss Axioscope) ^58^. % of area fibrosis was quantified using ImageJ software (National Institutes of Health, Bethesda, MD).

### Quantitative real-time polymerase chain reaction (RT-PCR)

Total mRNA was isolated from heart tissue using GeneJET RNA Purification Kit in RNase free condition following manufacturer’s instructions. The quality and quantity of RNA was evaluated by Nano Drop 2000 spectrophotometer (Thermo Fisher Scientific, MA, USA). 1 µg mRNA was reverse transcribed into complementary DNA (cDNA) using commercial RevertAid First Strand cDNA synthesis kit. Synthesized cDNA was used as the template for amplification and quantification of mRNA transcripts using CFX96 Touch™ detection system (Bio-Rad). Primers were designed using Primer3 online software and purchased from Sigma. 2X SYBR Green qPCR Master Mix was used for the PCR reaction assembly. The reaction was carried out at an initial heating step (95 °C for 1 min) followed by 40 repeats of amplification, that comprises denaturation at 95 °C for 5 s, and annealing at 60 °C for 30 s. The data was analyzed using CFX Manager ™ Software (Bio-Rad, Hercules, CA, USA) according to the manufacturer’s protocol. Transcript levels were quantified by normalizing to β-actin mRNA expression. The forward and reverse primer sequences used in this study are as follows:

**Table:** 

Gene	Type	Sequence
Superoxide dismutase (SOD)	Forward	5′-GCTCTAATCACGACCCACT-3′
	Reverse	5′-CATTCTCCCAGTTGATTACATTC-3′
Catalase (CAT)	Forward	5′-ATTGCCGTCCGATTCTCC-3′
	Reverse	5′-CCAGTTACCATCTTCAGTGTAG-3′
Glutathione peroxidase (GPx)	Forward	5′-CAGTTCGGACATCAGGAGAAT-3′
	Reverse	5′-AGAGCGGGTGAGCCTTCT-3′
Heme oxygenase-1 (HO-1)	Forward	5′-TGCTCGCATGAACACTCTG-3′
	Reverse	5′-TCCTCTGTCAGCAGTGCCT-3′
Transforming growth factor beta (TGF-β)	Forward	5′-AAGAAGTCACCCGCGTGCTA-3′
	Reverse	5′-TGTGTGATGTCTTTGGTTTTGTC-3′
β-Actin	Forward	5′- AGCCATGTACGTAGCCATCC-3′
	Reverse	5′- CTCTCAGCTGTGGTGGTGAA-3′

### Nuclear fractionation

We adapted nuclear fractionation protocol for rat cardiac tissues according to manufacturer’s instructions using the cell fractionation kit from Cell Signaling Technology. Briefly, small aliquot of cardiac tissue was subjected to successive fractionation steps for cytoplasmic, membrane and nuclear fractions. At the end of the fractionation steps, nuclear fraction was confirmed by the presence of the nuclear membrane marker lamin B1 by Western blotting as described in below.

### Cell culture and treatment

HL-1 cardiomyocyte cell line and cell culture components were purchased from Sigma Aldrich (St. Louis, MO, USA). HL-1 myocytes were cultured in Claycomb medium supplemented with 10% fetal bovine serum (FBS), 2 mM l-glutamine, 0.1 mM norepinephrine, 100 units/mL penicillin and 100 µg/mL streptomycin ^61^. The culture medium was replaced every 24 h. Cells were seeded at approximately 5,000/cm^2^ onto 0.02% gelatin/0.00125% fibronectin-coated dishes and grown to 80% confluency in the culture medium at 37 °C in a 95% air:5% CO_2_ humidified atmosphere. Experiments were conducted 12 h after removing FBS, antibiotics and norepinephrine to induce cells quiescence. For our experiments, HL-1 cells were treated as follows:

Control: treated with vehicle (DMSO).

Canaglif: treated with 10 µM canagliflozin for 12 h ^34^.

ISO: treated with 1 µM isoprenaline for 12 h ^62^.

ISO + Canaglif: co-treated with 1 µM isoprenaline and 10 µM canagliflozin for 12 h.

ISO + Canaglif + DM: co-treated with 1 µM isoprenaline and 10 µM canagliflozin for 11 h, and then with 10 µM dorsomorphin ^63^ for the last 1 h.

ISO + Canaglif + A443654: co-treated with 1 µM isoprenaline and 10 µM canagliflozin for 11.5 h, and then with 5 µM A443654 ^64^ for the last 30 min.

At the end of the treatment, cells were washed with ice-cold PBS, solubilized in RIPA buffer and processed for Western blotting as described below.

### Western blotting

About 0.1 g of heart tissue was cut into small pieces and lysed by Argos Tissue Homogenizer in RIPA buffer (50 mM Tris–HCl, 150 mM NaCl, 5 mM EDTA, 1% Nonidet P-40, 0.5% sodium deoxycholate, 0.1% SDS, 10 mM NaF, 10 mM Na_2_HPO_4_, pH 7.4) containing protease and phosphatase inhibitor cocktails. Tissue homogenates were centrifuged at 12,000 rpm for 15 min at 4 °C, supernatants collected and protein concentration determined using DC Protein Assay Kit. About 50 µg protein for each sample was boiled with 2X SDS-sample buffer (Bio-Rad) and resolved by SDS-PAGE. Resolved proteins were blotted onto PVDF membrane using BioRad semidry transfer apparatus. Where necessary, PVDF membranes were cut to allow simultaneous probing of multiple proteins without losing signal intensity of protein bands due to repeated stripping and reprobing. Membranes were then blocked with 5% milk solution in TBST (tris-buffered saline with 0.1% Tween 20) and incubated with the following primary antibodies overnight: total eNOS (Abcam, Cambridge, UK, 1:500 dilution), p-eNOS (Cell Signaling Technology, Danvers, MA, USA, 1:200 dilution), iNOS (Santa Cruz Biotechnology, Santa Cruz, CA, USA, 1:500 dilution), α-actin (Santa Cruz Biotechnology, 1:5,000 dilution), GAPDH (Santa Cruz Biotechnology, 1:5,000 dilution), Nrf2 (Cell Signaling Technology, 1:200 dilution), Lamin B1 (Santa Cruz Biotechnology, 1:100 dilution), HO-1 (Cell Signaling Technology, 1:100 dilution), TGF-β (Cell Signaling Technology, 1:100 dilution), p-Akt (Cell Signaling Technology, 1:400 dilution), total Akt (Cell Signaling Technology, 1:500 dilution), p-AMPK (Cell Signaling Technology, 1:400 dilution), total AMPK (Cell Signaling Technology, 1:500 dilution), cleaved caspase-3 (Cell Signaling Technology, 1:200 dilution) Bax (Santa Cruz Biotechnology, 1:200 dilution) and Bcl-2 (Santa Cruz Biotechnology, 1:200 dilution). Blots were then washed and incubated with HRP–conjugated secondary antibodies (Santa Cruz Biotechnology, 1:5,000 dilution) for 1 h at room temperature. At the end of secondary antibody incubation, blots were washed and developed using ECL (Pierce) and protein bands imaged using Gel Doc XR + System (Bio-Rad). Protein bands were quantified by densitometry using ImageJ software (64-bit Java 1.8.0_112, National Institutes of Health, Bethesda, MD; URL: https://imagej.nih.gov/ij/download.html). Blots were stripped of IgG using Restore™ stripping buffer and reprobed for further detection.

### Statistical analysis

For statistical analyses, OriginLab software version 9.55 (2018b, URL: https://www.originlab.com/index.aspx?go=SUPPORT&pid=3325) was used. Values were expressed as mean ± standard error of mean (SEM). One-way analysis of variance (ANOVA) along with Newman-Keuls post-hoc test was used for multiple comparison. p < 0.05 (a priori) was considered statistically significant.

## Supplementary information

Supplementary Information
