# Supplementary material for: Canagliflozin attenuates isoprenaline-induced cardiac oxidative stress by stimulating multiple antioxidant and anti-inflammatory signaling pathways
Source: Sci Rep. 2020 Sep 2;10:14459. doi: 10.1038/s41598-020-71449-1 (PMC7468124; doi:10.1038/s41598-020-71449-1)
Supplement: Supplementary file 1 — Supplementary Information [file 41598_2020_71449_MOESM1_ESM.pdf]

Supplementary material

Canagliflozin attenuates isoprenaline-induced cardiac oxidative stress by stimulating multiple antioxidant and anti-inflammatory signaling pathways

Raquibul Hasan[a*], Shoumen Lasker[b], Ahasanul Hasan[a], Farzana Zerin[a], Mushfera Zamila[b], Faizul Islam Chowdhury[b], Shariful Islam Nayan[b], Md. Mizanur Rahman[b], Ferdous Khan[b], Nusrat Subhan[b] and Md. Ashraful Alam[b*]

[a]Department of Pharmaceutical Sciences, College of Pharmacy, Mercer University, Atlanta, GA 30341, United States

[b]Department of Pharmaceutical Sciences, North South University, Dhaka 1229, Bangladesh

***Correspondence:**
Dr Md. Ashraful Alam, Associate Professor, Department of Pharmaceutical Sciences, North South University, Bashundhara, Dhaka, 1229, Bangladesh.
E-mail: ashraful.alam@northsouth.edu

And

Dr Raquibul Hasan, Assistant Professor, Department of Pharmaceutical Sciences, College of Pharmacy, Mercer University, 3001 Mercer University Drive, Atlanta, GA 30341, United States.
E-mail: hasan_r@mercer.edu

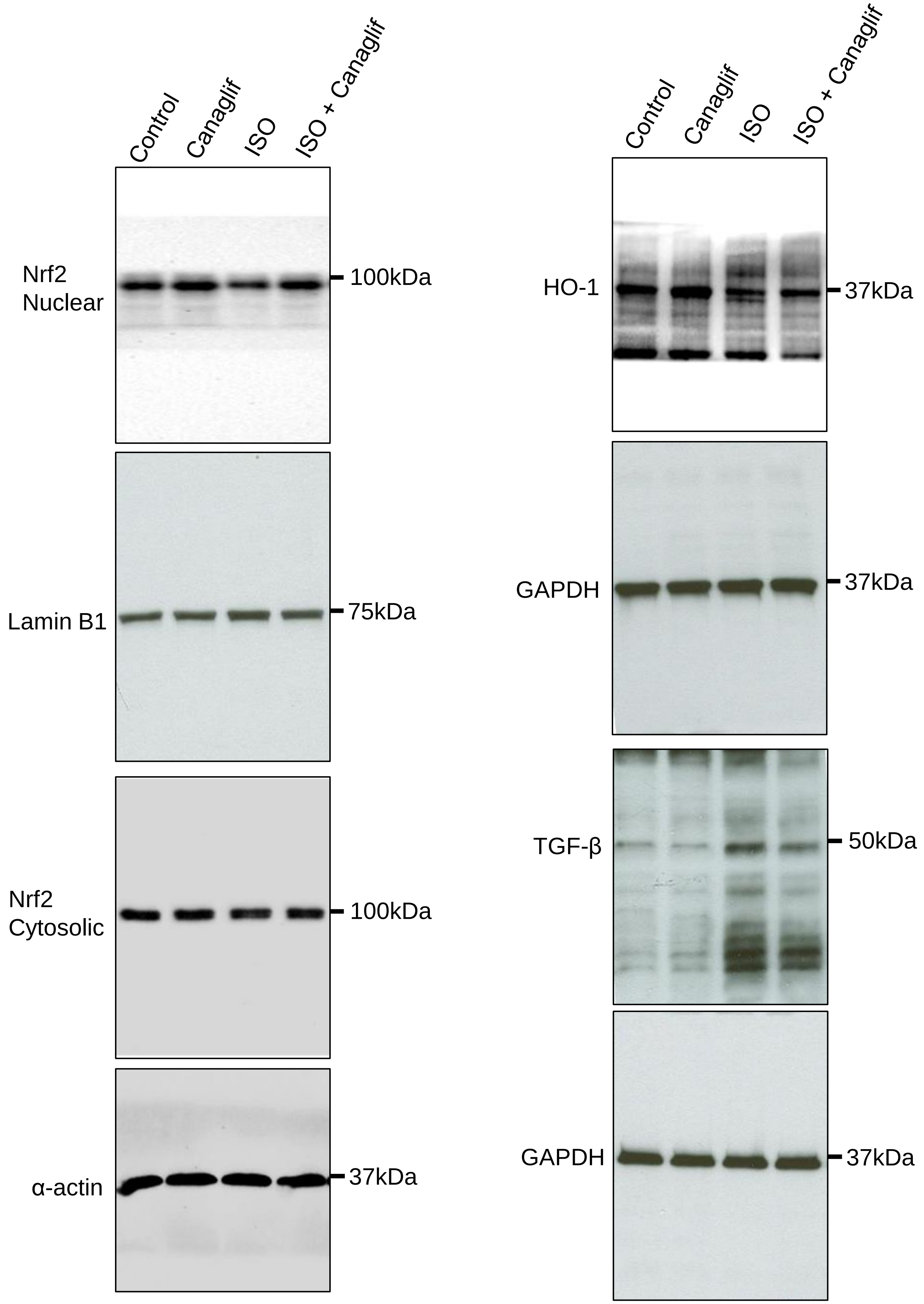

Supplementary Figure 1: Full-length Western blot images for Nrf2, Lamin B1, α-actin, HO-1, GAPDH and TGF-β representing Western blot images in Figure 5.

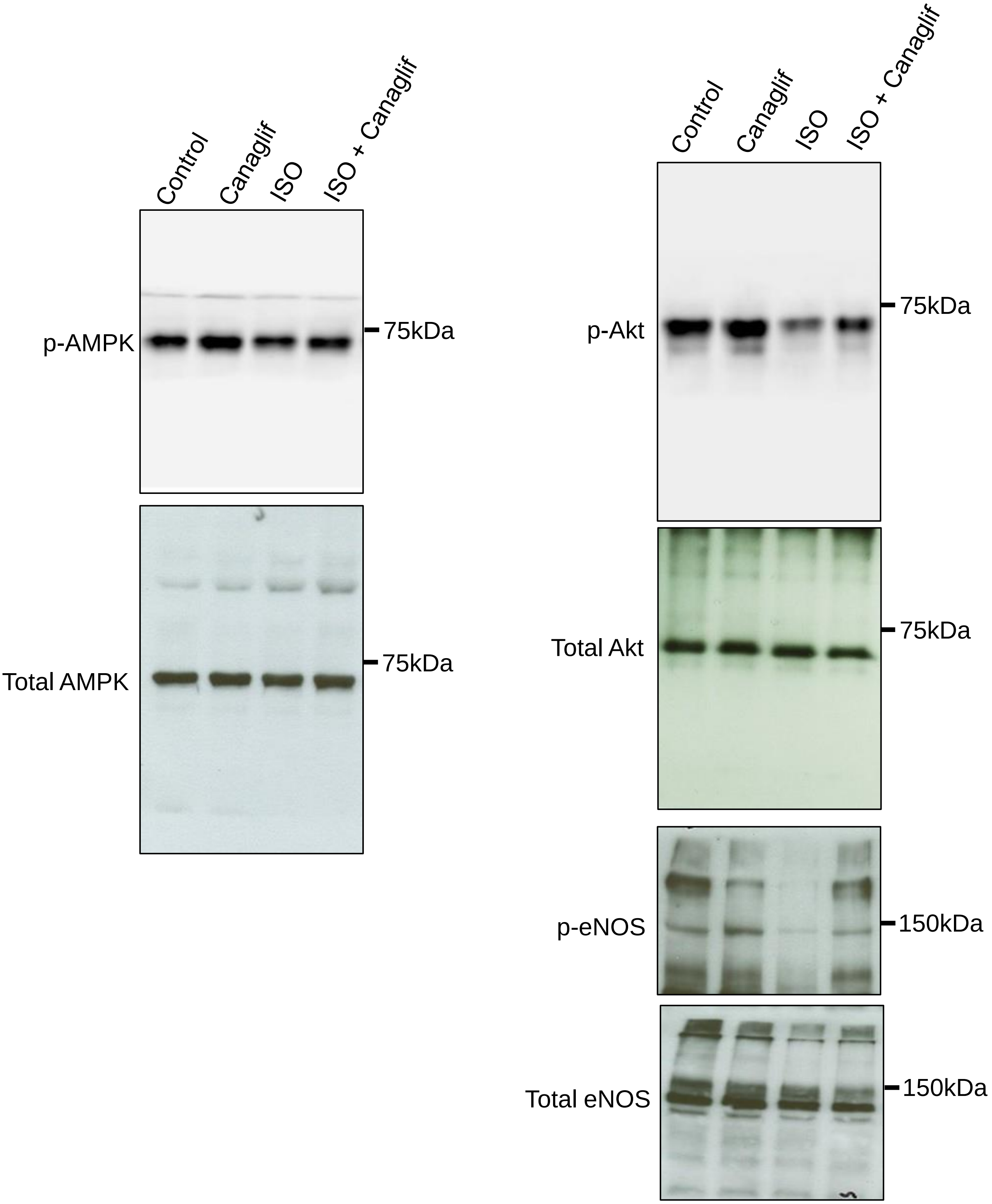

Supplementary Figure 2: Full-length Western blot images for p-AMPK, total AMPK, p-Akt, total Akt, p-eNOS and total eNOS representing Western blot images in Figure 6.

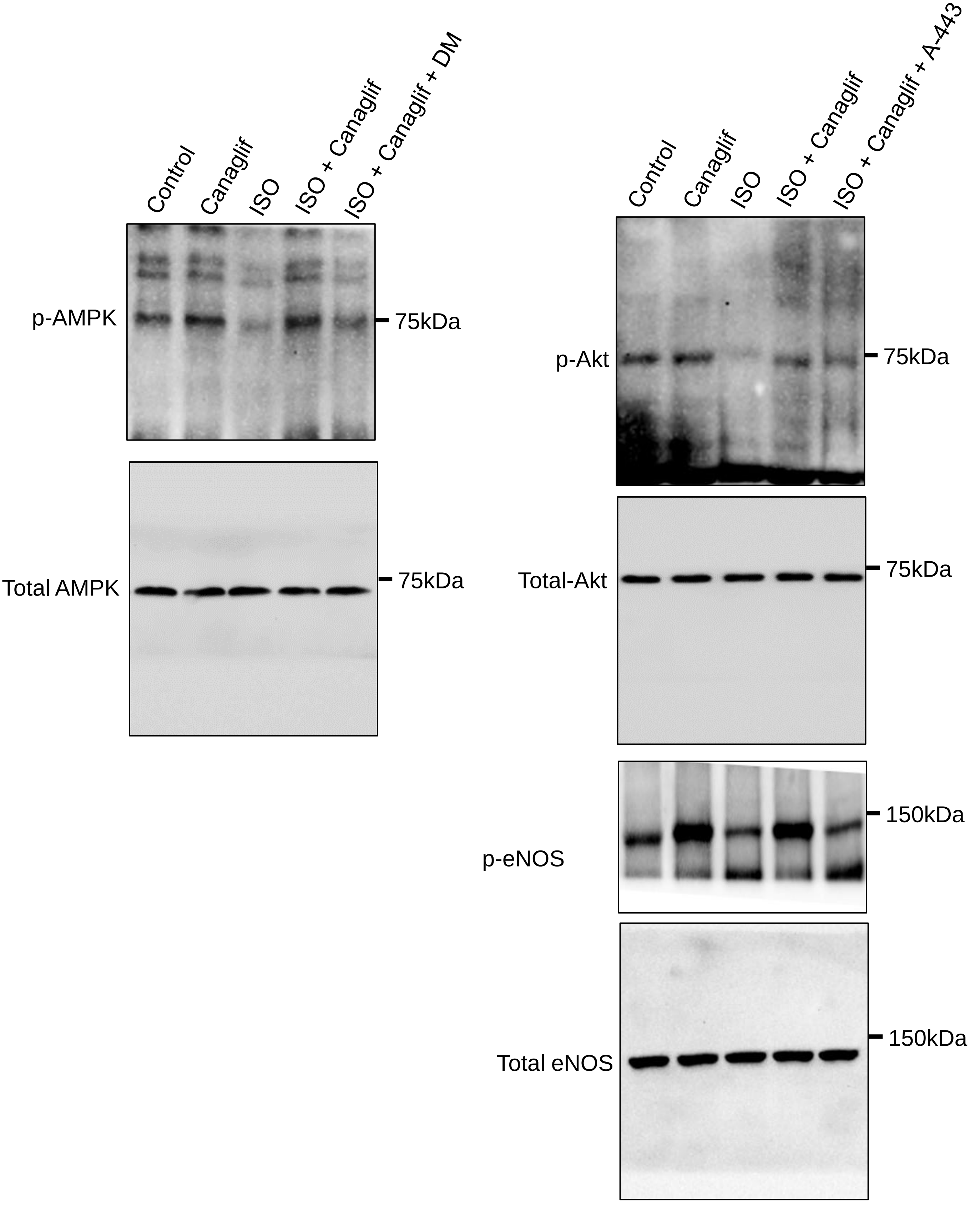

Supplementary Figure 3: Full-length Western blot images for p-AMPK, total AMPK, p-Akt, total Akt, p-eNOS and total eNOS representing Western blot images in Figure 7.

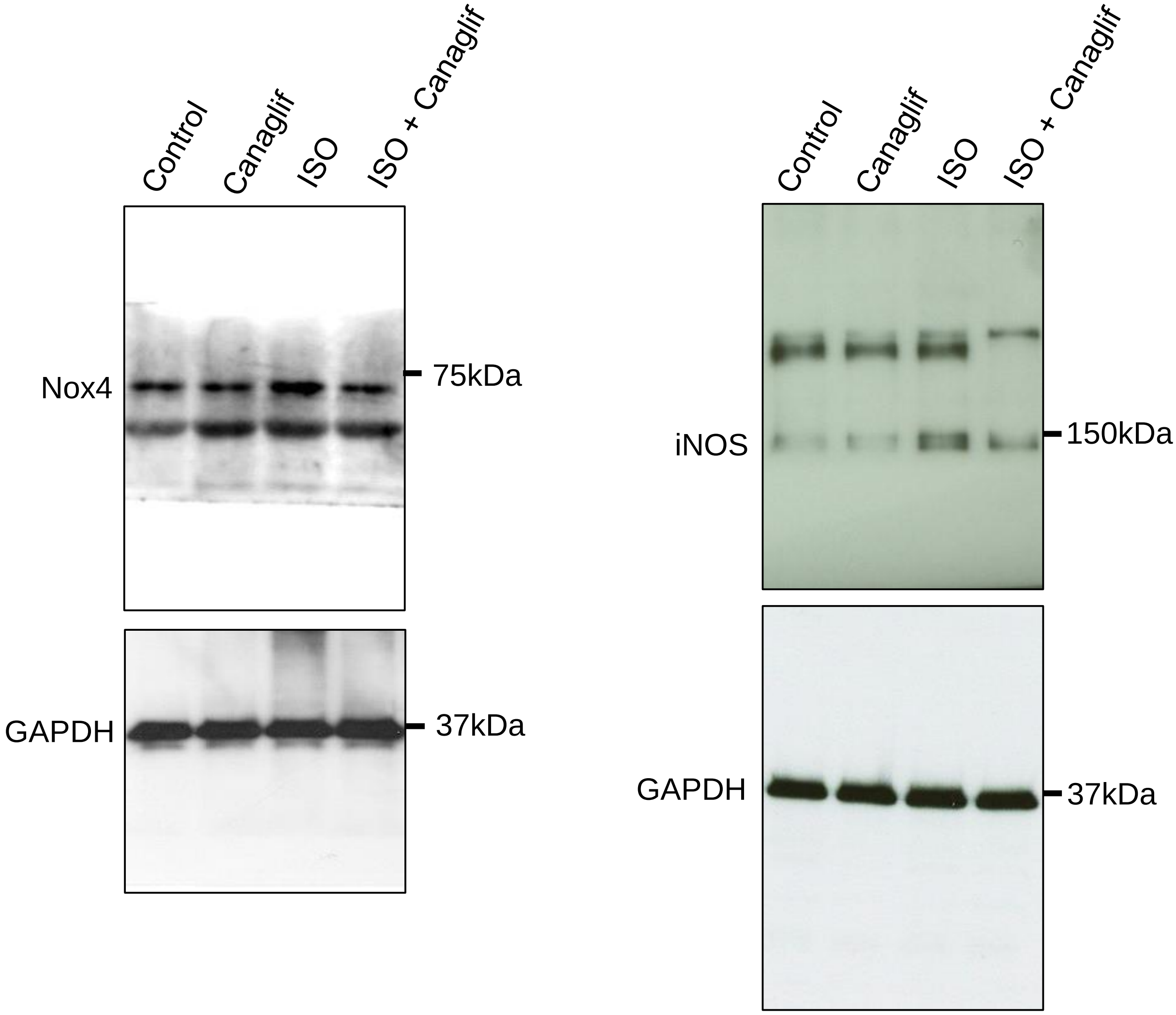

Supplementary Figure 4: Full-length Western blot images for Nox4, GAPDH and iNOS proteins representing Western blot images in Figure 8.

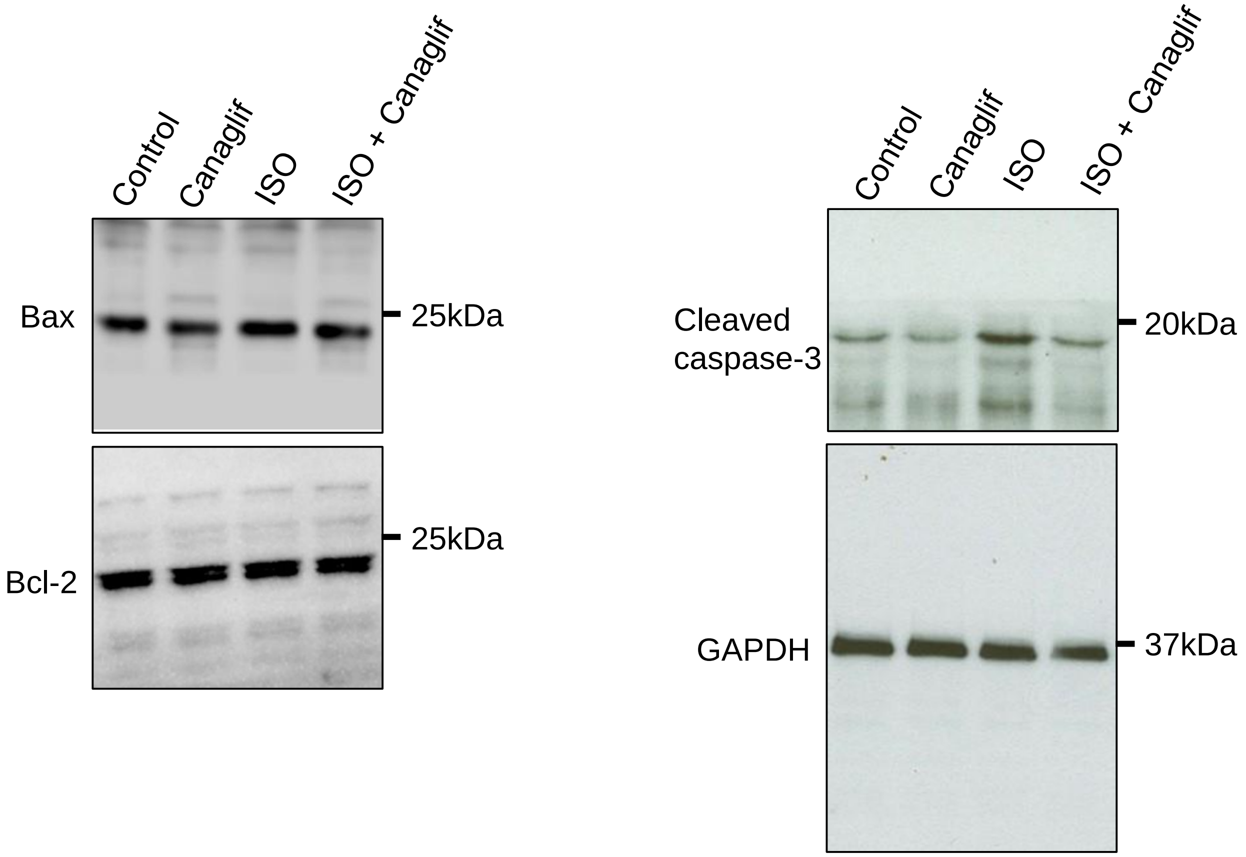

Supplementary Figure 5: Full-length Western blot images for Bax, Bcl-2, cleaved caspase-3 and GAPDH representing Western blot images in Figure 9.
